# Characterization of cuproptosis signature in clear cell renal cell carcinoma by single cell and spatial transcriptome analysis

**DOI:** 10.1007/s12672-024-01162-2

**Published:** 2024-07-24

**Authors:** Xiaohong Zou, Xiaoqing Liu, Huiting Wang, Zhenhua Li, Chen Zhou

**Affiliations:** Department of Laboratory Medicine, The Eighth Affiliated Hospital, Sun Yat-Sen University, Shenzhen, 518033 China

**Keywords:** Cuproptosis, Clear cell renal cell carcinoma, Single cell RNA sequencing, Spatial transcriptome, Immunosuppressive tumor microenvironment

## Abstract

Cuproptosis is a novel type to regulate cell death with copper-dependent manner, and has been reported to involve in the occurrence and development of various malignant tumors. However, the association between cuproptosis and the tumor microenvironment (TME) of clear cell renal cell carcinoma (ccRCC) remained unclear. To address this question, we integrated the single cell RNA sequencing (scRNA-seq) datasets of ccRCC across different stages, systematically examined the distinctive expression patterns of cuproptosis-related genes (CRGs) within the TME of ccRCC, and explored the crucial signatures using the spatial transcriptome sequencing (ST-seq) dataset. The cuproptosis activities reduced in cancer tissues along with the ccRCC development, and recovered after therapy. We identified *HILPDA*+ ccRCC1 subtype, characterized with hypoxia, as cuproptosis susceptible cells associated with a better prognosis. The main co-expression modules of *HILPDA*+ ccRCC1 subtype highlighted the role in anion transport, response to oxygen species and PD-L1-PD-1 pathway. Furthermore, the immunosuppressive cells might interact with *HILPDA*+ ccRCC1 subtype via HAVCR2-LGALS9, C3-C3AR1, HLA-A-CD8B and HLA-C-CD8A axises to shape the cuproptosis-related TME landscape. In summary, we anticipate that this study will offer valuable insights and potential strategies of cuproptosis for therapy of ccRCC.

**Graphical Abstract:**

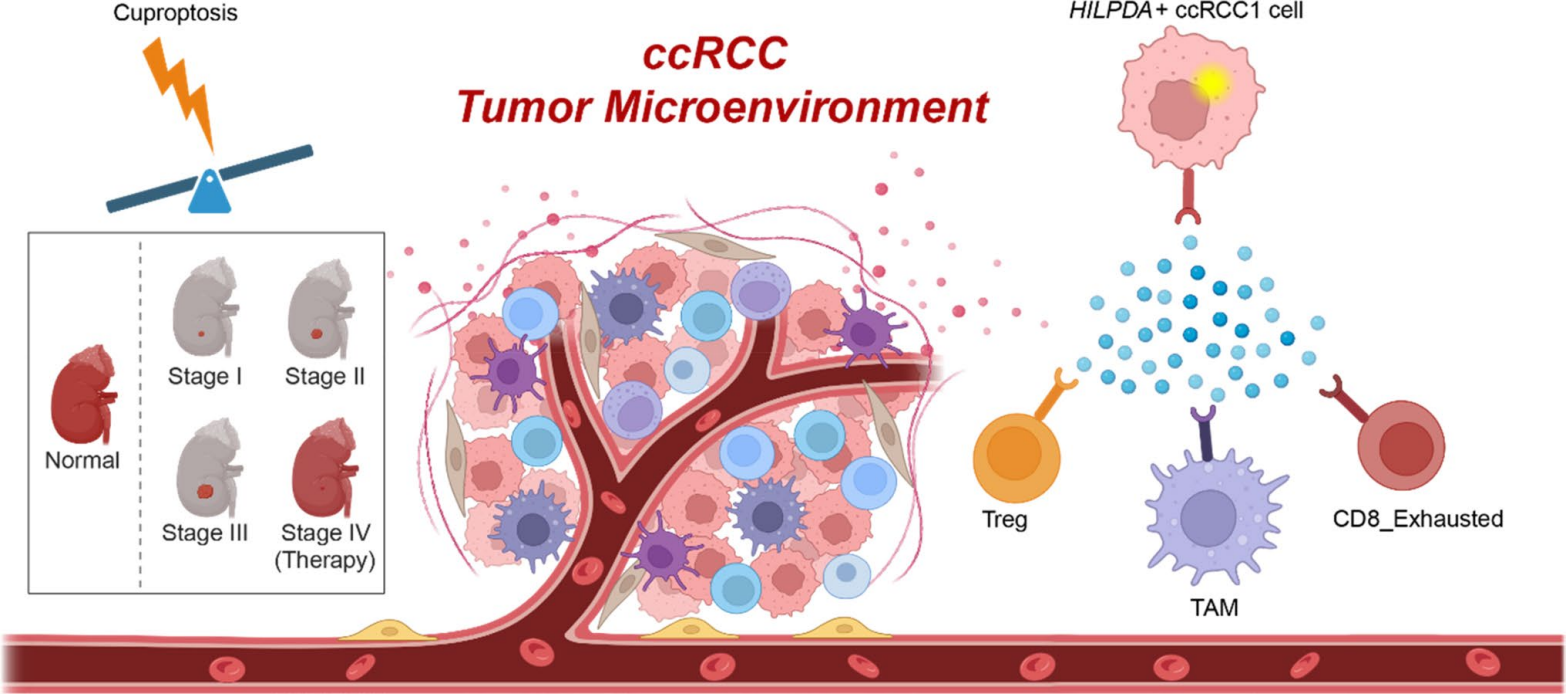

**Supplementary Information:**

The online version contains supplementary material available at 10.1007/s12672-024-01162-2.

## Introduction

Clear cell renal cell carcinoma (ccRCC) is the most common histological subtype of renal cell carcinoma (~ 75%) originating from renal tubular epithelial cells [1, 2], characterized with heterogeneous morphology and molecular attributes [3]. The most prominent feature of ccRCC is the presence of clear cytoplasm, attributable to the lipid and glycogen accumulation [4, 5], indicating the potential role of metabolic activity in ccRCC progression. Actually, ccRCC is essentially a metabolic disease marked by energetic metabolism reprogramming [6], including the increased glycolysis and lipid metabolism [7–10] in a grade-independent manner [11], enhanced pentosephosphate pathway (PPP) [12], as well as the impaired mitochondrial bioenergetics and oxidative phosphorylation (*OxPhox*) processes [13], and the downregulated tricarboxylic acid (TCA) cycle [14]. In addition, ccRCC is notable for its highly immune-infiltrated tumor microenvironment (TME) [15], which includes the enriched immune cells [i.e. B cells, defected CD8+ T cells [16], myeloid-derived suppressor cells (MDSCs), tumor associated macrophages (TAMs) [17]] and non-immune cells [i.e., cancer-associated fibroblasts (CAFs)] [18], as well as extracellular elements [19], showing an overall pro-inflammatory and angiogenesis profile [20]. In this scenario, complement system could be activated to facilitate immuosuppression [19, 21], and the reactive immunoflogosis could further drive cancer progression with an influx of cytokines and growth factors [22, 23]. More importantly, metabolism reprogramming crosstalk with anti-tumor immune response [18, 23, 24], shaping the immunometabolic rewiring among various immune cells during ccRCC development [25], ultimately promoting the tumor survival and growth.

Usually, ccRCC presents with an advanced stage of progression upon diagnosis. The patients in stage I exhibit a nearly 90% of 5-year survival rate, whereas in stage IV, it drops to less than 10% [26, 27]. At present, the general treatments for ccRCC cover immunotherapy, radiation, surgery and targeted therapy [28], including anti-angiogenic agents (sorafenib, sunitinib, pazopanib, axitinib and bevacizumab) [17], mTOR (temsirolimus and everolimus), and immune checkpoint inhibitors (nivolumab) [23]. However, the side effects and drug resistance significantly limit therapeutic efficacy, leading to a poor prognosis [29]. Therefore, it is immense clinical significant to explore the molecular mechanisms during ccRCC progress for the therapy development.

- Cuproptosis is a novel type of regulated cell death (RCD) that first identified by Tsvetkov et al. [30]. In the normal condition, copper acts as a cofactor for a variety of key metabolic enzymes [31], participate in energy conversion, signaling transduction, cell proliferation and angiogenesis; While the imbalanced copper level could impair mitochondrial respiration, leading to the dynamic changes of glycolysis and lipid metabolism [32]. Distinct from the oxidative stress-related cell death (such as apoptosis [33], ferroptosis [34], pyroptosis [35], disulfidptosis [36] and necrosis [37]), cuproptosis is associated with mitochondrial stress and shrinkage in a copper-dependent manner [38]. Mechanistically, the excess copper combined with the lipoylated proteins involved in TCA of mitochondria, leading to the agglomeration of lipoylated proteins and the instability of Fe-S cluster proteins, resulting in proteotoxic stress and ultimately cell death [39, 40].
- Cuproptosis is regulated by the altered metabolic factors and signaling pathways, such as abnormal copper accumulation, and disruptions in mitochondrial and lipoic acid metabolism [41]. Thereinto, copper overload could lead to reactive oxygen species (ROS) generation and cause oxidative stress, then impair the components and function of mitochondria, resulting to the reduced production of ATP and energy metabolism [42, 43]. Cuproptosis could also modulate immune cell infiltration and immunoflogosis. Cuproptosis-related genes (CRGs), such as *FDX1*, are associated with the infiltration of CD8^+^ T cells, and its overexpression can promote the infiltration to enhance the immune response, which are crucial for targeting and destroying cancer cells [44]. Patients with higher cuproptosis scores exhibited more significant immune cell infiltration, including CD8+ T cells and macrophages, which are crucial for anti-tumor immunity [45]. However, the mechanisms by which cuproptosis affects immune responses are not fully understood.

Previous studies have demonstrated that cuproptosis plays a crucial role in the occurrence and development of ccRCC [46]. Patients with ccRCC exhibit significant differences in clinical characteristics, immune cell infiltration and biological processes depending on the expression patterns of different CRGs [47]. These genes are generally downregulated in ccRCC compared with the normal tissues [48] and closely linked to patient prognosis [49], suggesting that they could serve as biomarkers for ccRCC progression. As mentioned previously, cuproptosis is associated with the tumor-infiltrating lymphocytes (TILs) in ccRCC, with higher expression levels of effector gene observed in group with higher cuproptosis score [49]. Risk scores constructed using CRGs can predict the infiltration levels of various immune cells in ccRCC, which are positively correlated with B cells, CD8+ T cells, natural killer cells, and negatively correlated with eosinophils, mast cells, and neutrophils [50]. Morever, cuproptosis status can guide the treatment of ccRCC patients. Patients with lower expression group respond better to PD-1 inhibitors than those with higher expression [48]. For patients with lower cuproptosis scores, conventional targeted therapy combined with guideline-recommended immunotherapy can be used as the first-line treatment. In contrast, for those with higher scores, a triple-drug combination of axitinib, pembrolizumab, and transforming growth factor-beta 1 (TGFB1) inhibitors is recommended [51]. The biochemical pathways involved in cuproptosis of ccRCC are being elucidated, however, the precise molecular mechanisms remain under debate. Furthermore, the therapeutic targeting of cuproptosis in ccRCC is controversial, and more research is needed to determine whether manipulating copper levels can be a viable treatment strategy without causing systemic toxicity. Thus, the deeper exploration of cuproptosis in ccRCC is necessary to reveal its biological involvement and improve the potential therapeutic effect.

Single cell RNA sequencing (scRNA-seq) could construct the transcriptomic characteristics of the individual cells, and obtain refined subpopulations to reveal the cellular heterogeneity of patients [52]. Meanwhile, spatial transcriptome sequencing (ST-seq) provides the cellular transcriptomic status in situ with spatial coordination [53], which is lost in scRNA-seq. In this study, we explored the cuproptosis-related TME dynamics across various stages of ccRCC progression, help to understand the role of cuproptosis in ccRCC and provide potential strategies for therapy.

## Materials and methods

### Data acquisition and processing

The single cell RNA sequencing (scRNA-seq) dataset (GSE210038 [54] and GSE207493 [55]) were obtained from GEO database, including 2 normal adjacent tissue (NAT) samples and 26 clear cell renal cell carcinoma (ccRCC) samples. R package Seurat (version 4.3.0) [56] was used to generate scRNA-seq object. Firstly, high-quality cells with (1) feature numbers (nFeature_RNA) ranged from 500 to 5000; (2) unique molecular identifier (UMI) counts (nCount_RNA) more than 500; (3) mitochondrial gene percentage less than 10%, were remained for further analysis. We then normalized and scaled data by *NormalizeData* and *ScaleData* function, and principal component analysis (PCA) was performed for linear dimensional reduction. Next, R package harmony (version 1.0.3) [57] was used to avoid batch effects from samples by *RunHarmony* function. We constructed a K-nearest neighbor (KNN) graph to neighborhoods and applied louvain algorithm to cluster cells by *FindNeighbors* and *FindClusters* function. Finally, non-linear dimensional reduction was performed by *RunUMAP* function to group similar cells together in low-dimensional space. In addition, 52 cuproptosis related genes (CRGs) were collected from previous studies [58, 59], including pro-cuproptosis CRGs such as *FDX1*, *LIAS*, *LIPT1*, *DLD*, *DLAT*, *PDHA1*, *PDHB*, and anti-cuproptosis CRGs *MTF1*, *GLS*, *CDKN2A*, et al. The CRGs scores were calculated by *AddModuleScore* function.

The spatial transcriptome sequencing (ST-seq) dataset was obtained from Mendeley Data platform (https://data.mendeley.com/datasets/g67bkbnhhg/1) and input to python environment. Geneset scores were calculated by *scanpy.tl.score_genes* function based on the cluster-specific genes, and visualized by *scanpy.pl.spatial* function.

### Differentially expressed genes identification and pathway enrichment analysis

We identified differentially expressed genes (DEGs) among different stages and cell types by *FindMarkers* function with adjusted P value < 0.05 and |logFC| > 0.25. Then biological processes from gene ontology (GO) dataset were enriched by R package clusterProfiler (version 4.8.1) [60]. Meanwhile, we performed gene set enrichment analysis (GSEA) [61], which could overcome the information bias caused by the hard threshold of DEGs and comprehensively evaluate changes of gene expression under different stages, to evaluate the pathway activities based on the sorted and unfiltered genes.

### Copy number variation inference

InferCNV [62] is used to identify large-scale chromosomal copy number variation (CNV) of somatic cells in tumor scRNA-seq data, including amplifications and deletions of whole chromosomes or large segments of chromosomes. Comparing with the reference set of Normal cells, the gene expression at different locations of each tumor cell were explored to find out chromosomal alterations. Epithelial cells were subset and were calculated by R package infercnv (version 1.12.0) with the default parameters to identify the real cancer cells.

### Survival analysis of HILPDA+ ccRCC1 cells in KIRC dataset

The cluster-specific genes of *HILPDA*+ ccRCC1 cells were subjected to GEPIA2 (http://gepia2.cancer-pku.cn). We used overall survival (OS) methods and set median of signatures as cutoff, finally obtained the significant survival outcome of *HILPDA*+ ccRCC1 genes in kidney renal clear cell carcinoma (KIRC) dataset.

### Identification of transcription factor regulon

We identified the main transcription factor (TF) by pySCENIC (version 0.12.1) [63], a computational method for simultaneous gene regulatory network reconstruction and cell-state identification from scRNA-seq data. Briefly, the count expression matrixs of Epithelial cells were exacted and generated the gene co-expression network by *grn* function. Using pre-computed database of cisTargetDB, we identified the cis-regulatory motifs by *ctx* function. Finally, the activities of each regulon were scored through AUCell algorithm by *aucell* function. To obtain the main regulons that potentially controlled transcription programs, we calculated the regulon specificity score (RSS) and visualized by *regulon_specificity_scores* function.

### Weighted gene co-expression network analysis

We used R package hdWGCNA (version 0.2.24) [64] to establish co-expression network and identify the hub gene modules of *HILPDA*+ ccRCC1 cells. Firstly, we constructed metacells from Epithelial cells to reduce data sparsity via the k-nearest neighbors (KNN) algorithm, and then selected soft power threshold as 10 by *TestSoftPowers* function, to generate the co-expression network by *ConstructNetwork* function. Next, we computed module eigengenes (MEs) and connectivity to obtain hub genes by *GetHubGenes* function, and visualized by *ModuleNetworkPlot* function. In addition, we calculated the correlations between CRGs score and MEs by *ModuleTraitCorrelation* function.

### TDEseq analysis

To find out the DEGs with temporal dynamic expression patterns in ccRCC development and progress, we performed R package TDEseq (version 1.1) [65] to Immune cells by *tdeseq* function. The four expression patterns including growth, recession, peak and trough could be visualized by *PatternHeatmap* function.

### Cell-cell communication analysis

The cell-cell communication network was constructed by R package CellChat (version 1.5.0) [66]. Generally, the expression matrix with subtype identifies was input to create a cellchat object by *createCellChat* function. Using CellChatDB.human as reference database, we computed the communication probability of the inferred ligand-receptors among different subtypes by *computeCommunProb* function and visualized by *netVisual_bubble* funtion.

### Statistical analysis

All statistical analyses were performed in R software (version 4.2.0), and adjusted *P* < 0.05 value was considered statistically significant unless otherwise specified.

## Results and discussions

### Single cell transcriptome atlas of ccRCC identifies the main cell types

**Fig. 1:**
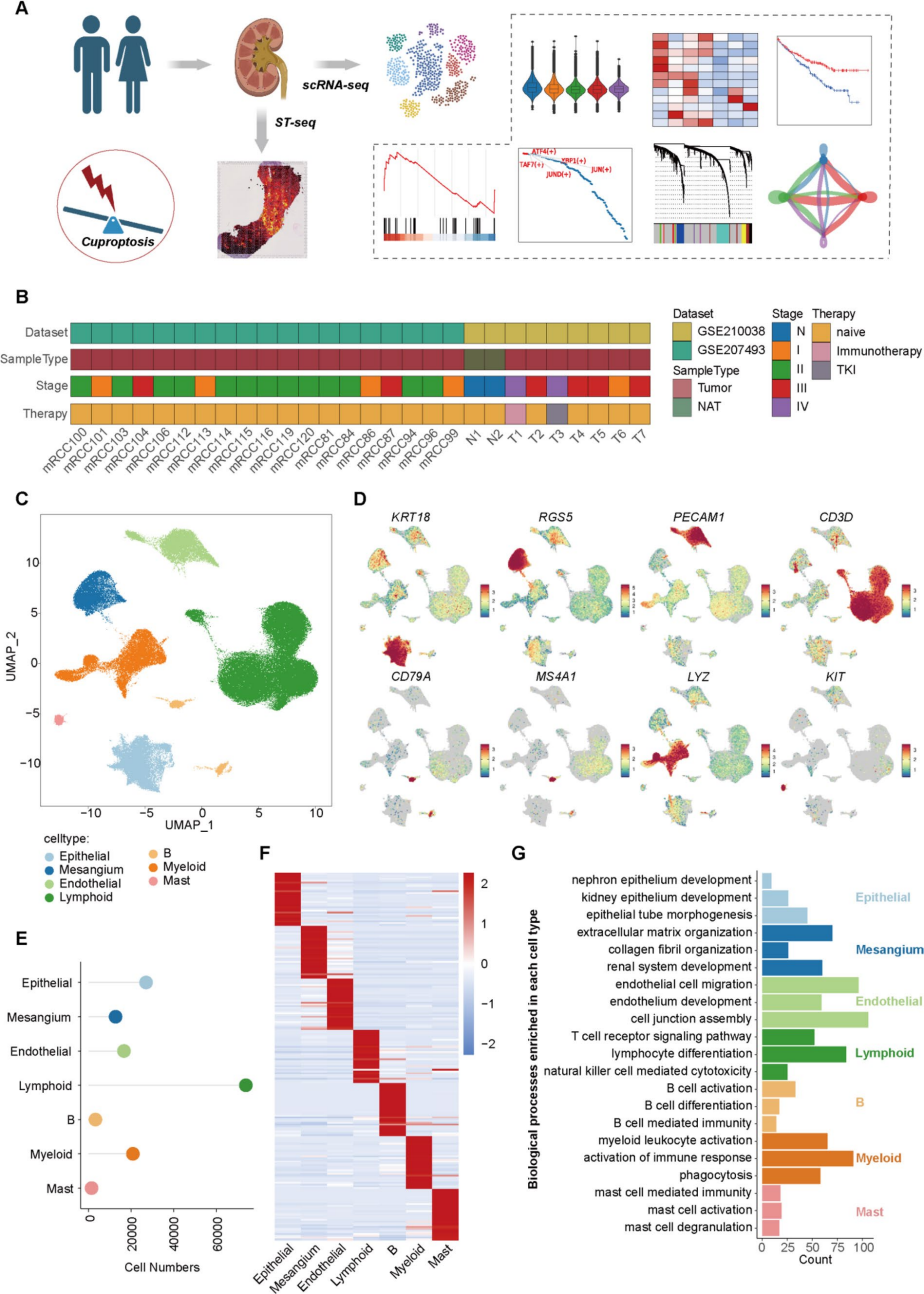
Cell Type Identification by Single cell RNA Sequencing dataset of ccRCC samples.  **A** The workflow of this study; **B** the sample information of scRNA-seq dataset; **C** UMAP showing 7 main cell types based on scRNA-seq dataset; **D** the relative expression of canonical markers in each cell type; **E** lollipop chart showing cell numbers of each cell type; **F** heatmap showing cluster-specific genes of each cell type; **G** bar plot showing the enriched biological processes of each cell type.

The workflow in this study was shown as Fig. 1A. Firstly, we collected single cell RNA sequencing (scRNA-seq) data of 28 clear cell renal cell carcinoma (ccRCC) samples from the two independent datasets (GSE210038 [54] and GSE207493 [55]) (Fig. 1B), including 2 samples of normal adjacent tissue (N), 5 of stage I (I), 13 of stage II (II), 6 of stage III (III) and 2 of stage IV (IV). It’s worth noting that samples from stage IV were treated with immunotherapy and tyrosine kinase inhibitors (TKI) and showed good response. We obtained 155,399 cells after quality control (Fig S1), and identified 7 main cell types based on the relative expression of canonical markers (Fig. 1C, D), including Epithelial (*KRT18*+), Mesangium (*RGS5*+), Endothelial (*PECAM1*+), Lymphoid (*CD3D*+), B (*CD79A*+), Myeloid (*LYZ*+), Mast (*KIT*+), which showed un-batched among different samples (Fig S2). Thereinto, Lymphoid cells occupied the most numbers of total (*n* = 73,906, 47.56%), followed by Epithelial (*n* = 26,981, 17.36%) and Myeloid (*n* = 20,790, 13.38%) (Fig. 1E). Furthermore, we identified the cluster-specific genes and performed the biological processes enrichment (Fig. 1F, G, Table S1), which revealed the features and functions of each cell type. For example, Epithelial cells were associated with kidney epithelial development, Mesangium cells participated in collagen fibril organization, Lymphoid cells enriched in T cell receptor signaling pathway. Therefore, we successfully constructed the single cell transcriptome atlas of ccRCC for the follow-up analysis.

### Characterization of transcription programs during ccRCC development

**Fig. 2:**
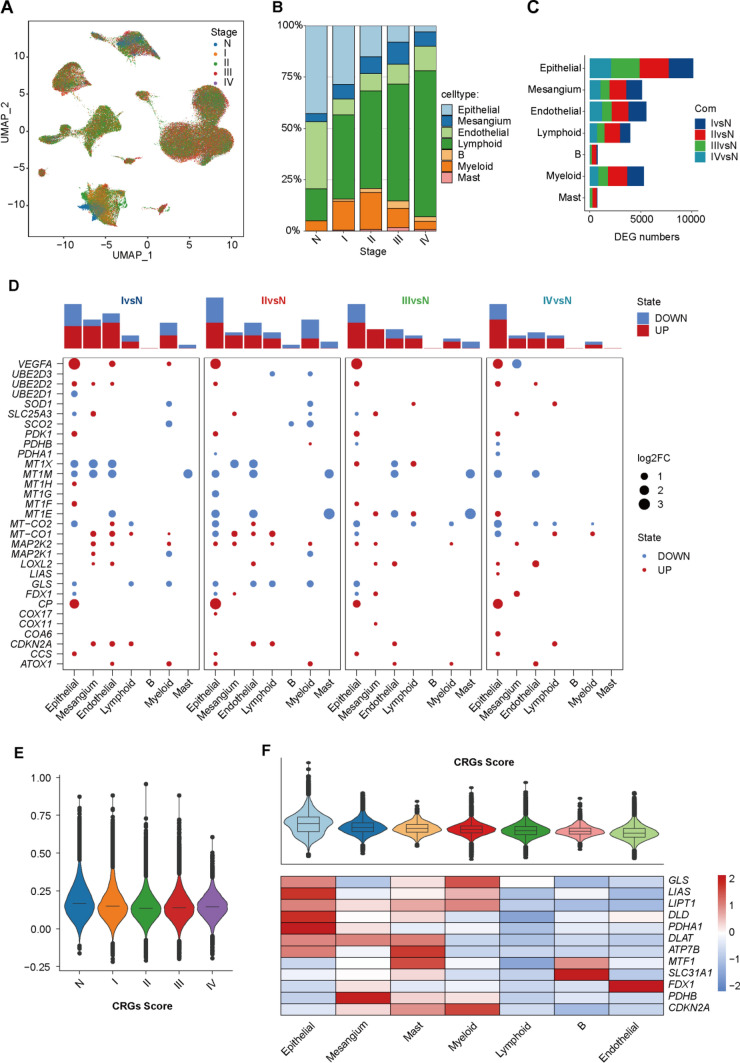
Dynamics changes in the transcriptional profiles during ccRCC progress.  **A** UMAP showing the distribution of cells from different stage; **B** bar plot of the relative cellular proportions of cell types in each group; **C** the number of DEGs between tumor groups and normal group in each cell type; **D** dot plot showing the differentially expressed CRGs in each cell types across different stages. The total numbers were listed to top panel; **E** violin plot showing the relative CRGs score in each stage; **F** violin plot showing the relative CRGs score in each cell type (top) and heatmap showing the relative expression levels of CRGs in each cell type (bottom).

Next, we explored the cellular composition and gene expression changes of different stages along with the ccRCC development. The main cell types were shared among different stage identities (Fig. 2A), albeit at different proportions, in which Immune cells infiltration was elevated in tumor samples (Fig. 2B), as evidenced by a larger percentage up to 56.62%, 68.18%, 71.62%, 78.12% than that 20.56% of N samples, indicating the role of infiltrated immune cells during ccRCC development [67], thus we next explored the alteration of transcriptome programs among different stages except for the cell number changes. The differentially expressed genes (DEGs) between tumor and normal groups were identified, which showed the most abundance in Epithelial cells across the 4 stages (Fig. 2C), suggesting its significant response during ccRCC progress. The biological processes of each cell type at different stages showed generally disorder in ATP metabolic process, regulation of T cell activation, renal system development and so on (Fig S3, Table S2). Furthermore, we overlapped the cuproptosis-related genes (CRGs, Table S3) with stage-related DEGs, which displayed the widespread dysfunction (Fig. 2D, Table S4). For example, pro-cuproptosis *FDX1* [68] was down-regulated in stage I–III, while not significant in stage IV; and anti-cuproptosis *CP* [69] and *CDKN2A* [70] were up-regulated in tumor groups. In addition, Epithelial cell harbored the most overlapped DEGs compared with the other cell types. We next calculated the CRGs geneset score by *AddModuleScore* function, which gradually reduced in stage I–III and recovered in stage IV (Fig. 2E), indicating that cuproptotis were protective factors of ccRCC [48, 71] and therapy could enhance this effect. As shown in the DEGs distribution (Fig. 2D), Epithelial cells demonstrated the highest scores among the main cell types (Fig. 2F), and enriched key CRGs such as *LAIS*, *DLD*, *LIPT1* and *PDHA1*, in line with the previous studies that epithelial cells might be susceptible to target cuproptosis [45, 72]. Collectively, these results illustrated the decreased cuproptosis signature activities along with the ccRCC development, in which Epithelial cells were the most susceptible and showed the strongest response.

### *HILPDA*+ ccRCC1 subtype exhibits cuproptosis-susceptible in ccRCC

**Fig. 3:**
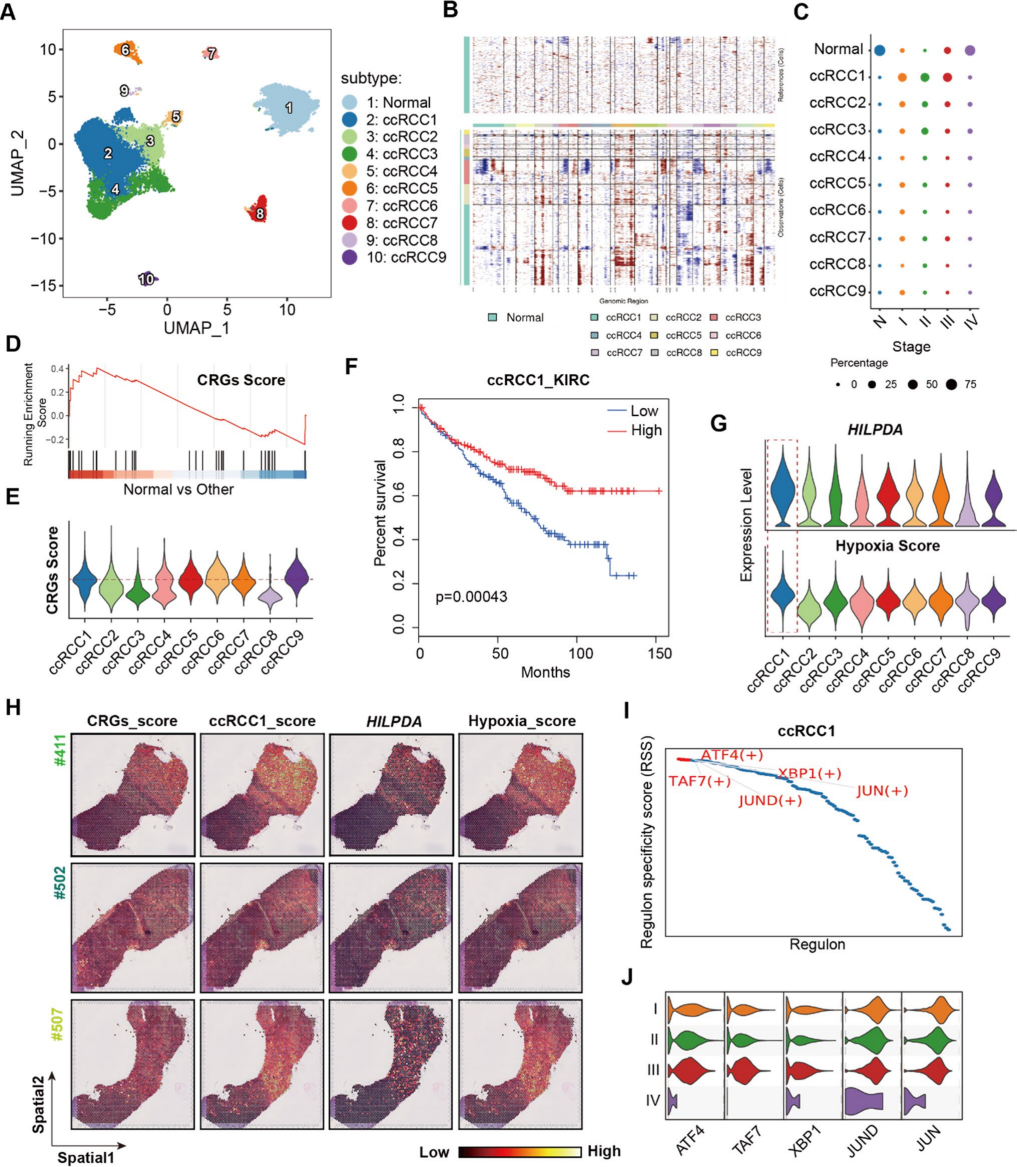
Transcription programs of ccRCC cells in response to cuproptosis. **A** UMAP showing the 10 subtypes of 26,981 Epithelial cells; **B** heatmap showing inferred CNV of scRNA-seq dataset; **C** dot plot of the relative cellular proportions of Epithelial subtypes in each group; **D** GSEA analysis revealed the activated CRGs enriched in Normal Epithelial cells; **E** violin plot showing the relative CRGs score in each cancer subtype; **F** survival plot of *HILPDA*+ ccRCC1 signature high and low group in the KIRC samples; **G** violin plots of *HILPDA* expression levels and hypoxia scores in each cancer subtypes; **H** spatial transcriptome displayed the distribution of CRGs, *HILPDA*+ ccRCC1 signatures, hypoxia scores and *HILPDA* expression; **I** the regulon specificity scores of TFs in *HILPDA*+ ccRCC1 subtype. The top 5 TFs ordered by scores were listed; **J** violin plot showing the expression levels of the top 5 TFs in *HILPDA*+ ccRCC1 subtype across stage I–IV.

To investigate the cellular heterogeneity of Epithelial cells response to curpotosis, we re-clustered them into 10 subtypes (Fig. 3A, Fig S4), including one type of Normal cells and 9 of cancer cells. The inferred copy number variation (CNV) scores of each cell (Fig. 3B) revealed that annotated cancer cells (ccRCC1-9) harbored more intense amplifications or deletions than Normal cells [73, 74]. To characterize the transcription programs of different subtypes, we enriched the relevant biological processes (Fig S5, Table S5) according the cluster-specific genes (Fig S4), and found that Normal subtype was related with epithelial cell migration and kidney development, ccRCC1 subtype was involved in hypoxia response and HIF-1 signaling pathway, and so on.

As expected, N group was enriched mostly with Normal cells, and stage I–III group with ccRCC1 cells, while the cellular proportion of Normal cells was restored in stage IV (Fig S6, Fig. 3C), highlighting the role of ccRCC1 cells in tumor progression. In contrast to other cell types, Normal cells show a higher CRGs score (Fig. 3D), while ccRCC1 within cancer cells exhibited relatively elevated scores (Fig. 3E), suggesting that lower CRGs score was strongly related with higher cancer stages [68]. Given the higher proportion of ccRCC1 cells in tumor groups and its increased susceptibility to cuproptosis, we next explored its prognosis outcome in kidney renal clear cell carcinoma (KIRC). We firstly subset cluster-specific genes of ccRCC1, and divided samples into high/low groups based on the median of the signature expression level. Results showed that low group were significantly associated with poor survival (Fig. 3F), indicating that the CRGs-enriched ccRCC1 cells could potentially serve as biomarkers for KIRC. Actually, multiple CRGs, such as *DLAT*, *SLC31A1*, *MTF1*, *ATP7B*, *FDX1*, *ATP7A*, and *DLD*, were correlated with the better prognosis and identified as protective genes for ccRCC patients [48].

We noticed that ccRCC1 cells were characterized by hypoxia inducible lipid droplet associated (*HILPDA*) gene (Fig S4, Fig. 3G), whose knockdown reduced tumor volume in the xenograft model, thus served as the potential therapeutic targets for ccRCC [75]. The expression of *HILPDA* could be induced by HIF1-α [76], whose accumulation was related with *VHL* gene mutation (a hallmark of ccRCC) [77]. Correspondingly, this remarkable subtype exhibited elevated hypoxia scores as well in the tumor microenvironment (TME) (Fig. 3G). Due to the lost spatial information during tissue dissociation of scRNA-seq dataset [78], we therefore evaluated the relationship of *HILPDA*+ ccRCC1 subtype with CRGs by spatial transcriptome sequencing (ST-seq). We obtained ST-seq dataset from 3 ccRCC patients (Fig. 1A), and scored the genesets of CRGs, *HILPDA*+ ccRCC1 subtype, hypoxia as well as the expression level of *HILPDA* (Fig. 3H). The co-location of these characteries were found in tumor tissue across the three samples, indicating the cuproptosis activity enriched in *HILPDA*+ ccRCC1 subtype. This identification approach has been applied to reveal the higher pyroptosis scores in immune cells of melanoma [79], tumor-specific keratinocyte in fibrovascular niche of squamous cell carcinoma [80].

In summary, we identified *HILPDA*+ ccRCC1 subtype as the most susceptible cancer cells to cuproptosis. We next performed single cell regulatory network inference and clustering (SCENIC) to find out the critical regulons that impacting the biological characteristics of *HILPDA*+ ccRCC1 subtype. According to the regulon specificity scores (RSS), TFs such as ATF4, TAF7, XBP1, JUND and JUN were the main regulons (Fig. 3I), which expression levels were also higher than that of stage IV group (Fig. 3J). Previous studies have verified that ATF4 contributes to transcriptional and metabolic remodeling in ccRCC [81], and XBP1 could cause immunosuppression to regulate the tumor growth [82].These results illustrated the importance of cuproptosis-susceptible *HILPDA*+ ccRCC1 subtype during cancer development.

**Fig. 4:**
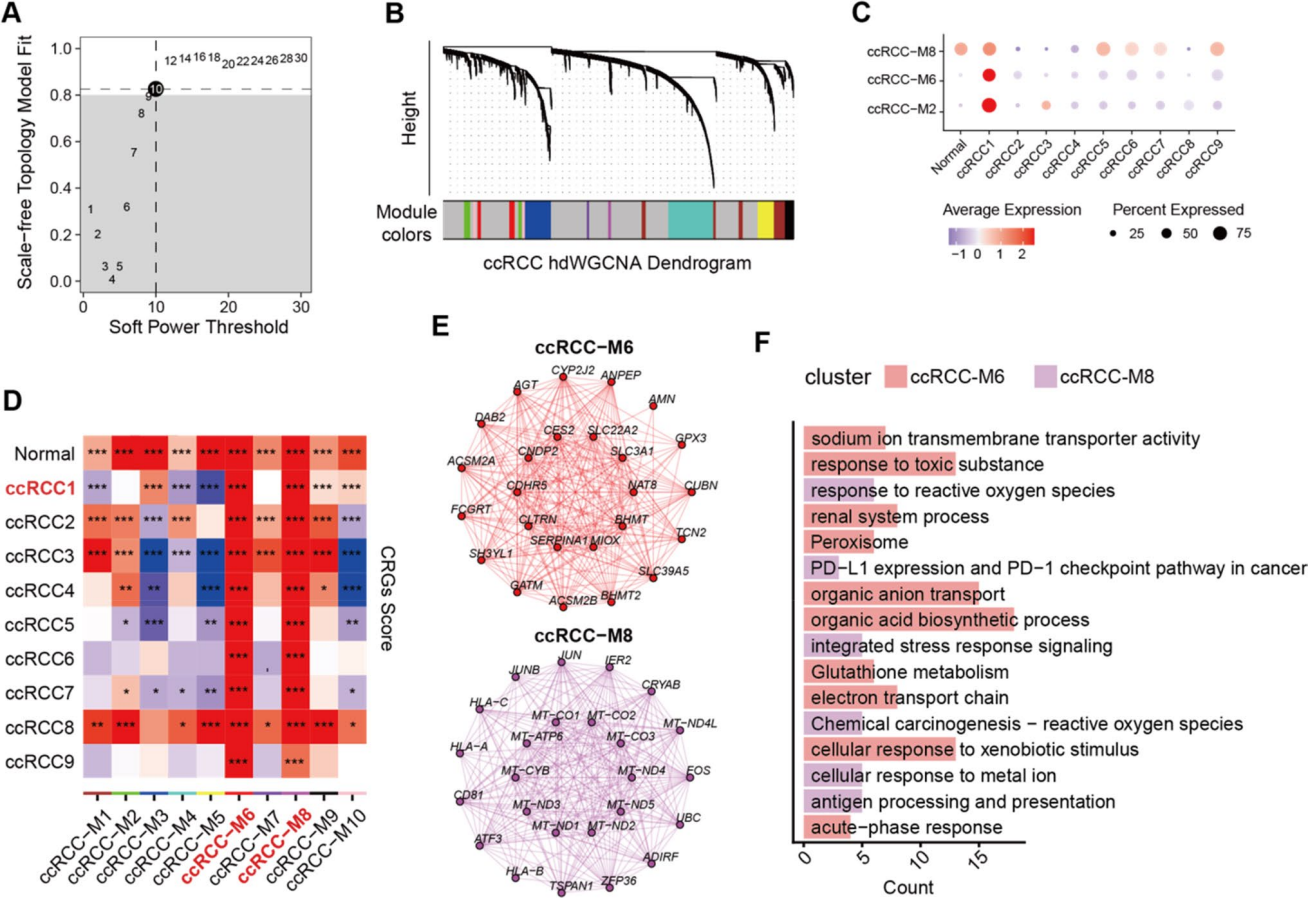
Weighted gene co-expression network of *HILPDA*+ ccRCC1 cells.  **A** Soft power threshold identification of Epithelial cells; **B** Dendrogram plot showing the gene modules of co-expression network; **C** dot plot indicating the average expression levels of gene modules across the different Epithelial subtypes; **D** heatmap showing correlation between the eigengenes of each module with CRGs across the subtypes; **E** the top hub genes within ccRCC-M6 and M8 modules; **F** bar plots of the biological processes enriched in ccRCC-M6 and M8 modules.

Furthermore, high dimensional weighted gene co-expression network analysis (hdWGCNA) was performed to reveal the intrinsic regulatory networks of *HILPDA*+ ccRCC1 subtype. We chose the soft power threshold as 10 to construct the co-expression network (Fig. 4A), and identified 10 significant gene modules (Fig. 4B). The signature expression levels of each module among Epithelial subtypes implied that ccRCC-M2, M6 and M8 were activated in *HILPDA*+ ccRCC1 subtype (Fig. 4C). In addition, the eigengenes of ccRCC-M6 and M8 were positively correlated to the CRGs score of *HILPDA*+ ccRCC1 subtype (Fig. 4D), emphasizing the overall expression enrichment of M6 and M8. The hub genes were shown as Fig. 4E, and mainly participated in organic anion transport [83], response to reactive oxygen species (ROS) [84], PD-L1 expression and PD-1 checkpoint pathway in cancer [85] (Fig. 4F), suggesting the significance tumor immune, thus we next explored the correlation between cuproptosis and TME.

### Immunosuppressive cells interact with *HILDPA*+ ccRCC1 cells in tumor microenvironment

**Fig. 5:**
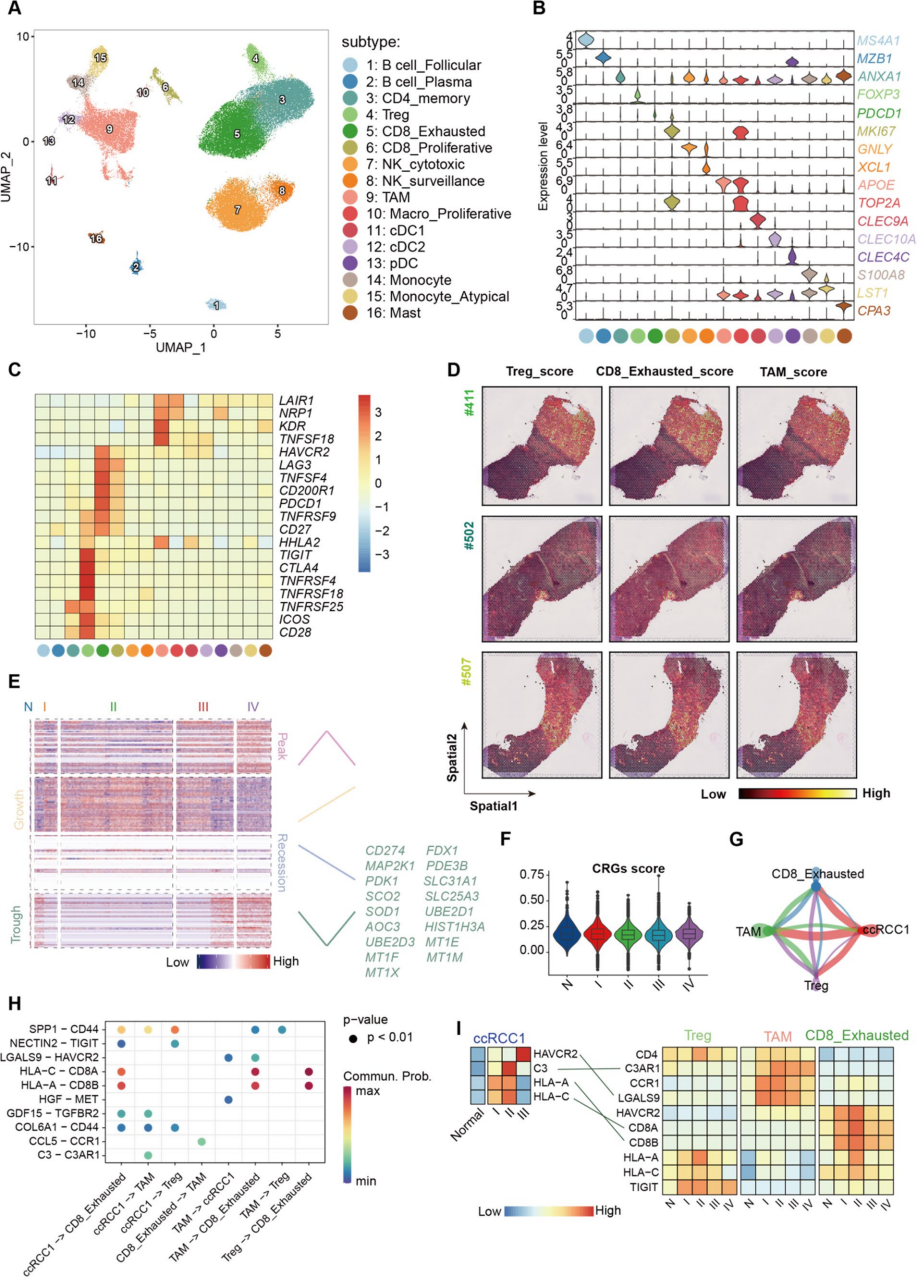
Dissection of immunosuppressive cells of cuproptosis-related tumor microenvironment. **A** UMAP showing the 16 subtypes of 99,210 Immune cells; **B** violin plot of the relative expression levels of the canocial markers in each subtype; **C** heatmap showing the enrichment of immune checkpoint and suppressive genes; **D** spatial transcriptome displayed the distribution of Treg, CD8_Exhausted and TAM signature scores; **E** heatmap showing the four gene expression patterns deduced by TDEseq analysis; **F** violin plots showing the relative expression levels of CRG scores in the immunosuppressive cells across different stages; **G** Chord diagram showing the number of interactions among the four subtypes; **H** Bubble plot showing the ligand-receptor pairs in the main subtype; **I** Heatmap showing the relative expression levels of key genes of the four subtypes among the different stages. The paired ligand-receptor shown in **H** were connected by lines.

To further characterize the immune profiles in the TME, we re-clustered Immune cells to obtain 16 subtypes (Fig. 5A), including B cell_Follicular (*MS4A1*+), B cell_Plasma (*MZB1*+), CD4_memony (*ANXA1*+), regulatory CD4+ T (Treg, *FOXP3*+), CD8_Exhausted (*PDCD1*+), CD8_Proliferative (*MIK67*+), NK_cytotoxic (*GNLY*+), NK_surveillance (*XCL1*+), tumor-associated macrophage (TAM, *APOE*+), Macro_Proliferative (*TOP2A*+), conventional dendritic cell 1 (cDC1, *CLEC9A*+), cDC2 (*CELC10A*+), plasmacytoid dendritic cell (pDC, *CLEC4C*+), Monocyte (*S100A8*+), Monocyte_Atypica (*LST1*+) and mast (*CPA3*+), which were characterisized by the canocial markers (Fig. 5B). Among these immune cells, we found that immune checkpoint and suppressive genes, such as *PDCD1*, *CTLA4*, *TNFRSF4* [45], were highly expressed in Treg, CD8_Exhausted and TAM subtypes (Fig. 5C), hinting their immunomodulatory effects in the TME. Likewise, we scored the cluster-specific genes of these immunosuppressive cells using ST-seq dataset, which were consistent with the spatial location with *HILPDA*+ ccRCC1 cells (Fig. 5D).

We next performed TDEseq analysis of immunosuppressive cells to find out the DEGs with temporal dynamic expression patterns along with the tumor progress. Focusing on the genes with the different four modules, we identified CRGs mostly appeared as trough tendency, such as *FDX1* and *SLC31A1* (Fig. 5E), in line with the distribution of CRGs scores among different stages (Fig. 5F).

The spatial co-location of immunosuppressive cells and *HILPDA*+ ccRCC1 subtype indicated the potential interaction among these cells in the cuprostosis-related TME. Thus, we applied cell-cell communication of these four subtypes (Fig. 5G) using scRNA-seq dataset, and found out several key ligand-receptor (LR) pairs. Immunoregulatory molecules, including complement component C3-C3AR1, chemokine CCL5-CCR1, major histocompatibility complex HLA-A/C-CD8A/B, as well as the inhibitor receptor LGALS9-HAVCR2 and NECTIN2-TIGIT (Fig. 5H), mediated the cellular interaction of TME. The relative expression levels of these genes were compared among the different stages of each subtype (Fig. 5I). We removed the *HILPDA*+ ccRCC1 cells of stage IV due to its low number (*n* = 3) and used the Normal epithelial cells from NAT samples as control. The results supported the potential relationship between *HILPDA*+ ccRCC1 subtype and immunosuppressive cells via HAVCR2-LGALS9, C3-C3AR1, HLA-A-CD8B and HLA-C-CD8A axises. HAVCR2, encoding immune inhibitor receptor TIM-3, could induce tumor immune escape when bind with LGALS9 [86], which played the main role in the cellular communication between TAM and the other cells [87]. Complement C3 derived by tumor cells could regulate TAMs via C3a-C3aR-PI3Kγ signaling to inhibit anti-tumor immunity [88, 89]. Taken together, these results emphasized the crucial role of immunosuppressive cells and their crosstalk in the cuproptosis-related TME.

As our results shown, *HILPDA*+ ccRCC1 harbored hypoxia characterization, which could attract Tregs [90], polarize macrophages toward M2-like phenotype [91], reduce the function of activated T cells [92], and directly promote the transcriptional activation of immunosuppressive factors [93]. In addition, the relative expression levels of *HILPDA*, which was regarded as the marker of ccRCC1 cells in our study, is positively correlated with TAM infiltration and expression of immunosuppressive genes such as *PD-L1*, *PD-1*, *HAVCR2*, *TGFB1* and *TGFBR1* [94], modulating TME as an immunosuppressive state. Thus, we inferred that *HILPDA*+ ccRCC1 cells could attract and promote the form of immunosuppressive cells in the hypoxia TME of ccRCC to promote tumor development.

Interestingly, the immunosuppressive TME could be reversed by cuproptosis. Xing et al. [95] firstly designed nanoparticles that can induce cuproptosis (NP@ESCu), then combined with αPD-L1 to reprogram the immunosuppressive TME, thereby enhancing the immunotherapy of bladder cancer. Shen et al. [96] also constructed nanoparticles (ES@CuO) to induce cuproptosis-based immunotherapy of melanoma. This therapy has been applied to other cancers (such as breast cancer [97, 98] and rectal cancer [99]). We believe that cuproptosis-targeted therapy aiming at *HILPDA*+ ccRCC1 subtype in our study could improve the immunosuppressive TME and promote the immunotherapy efficacy of ccRCC.

This study has several limitations that need to be considered. Firstly, the dataset is collected from the public studies, which might affect the generalizability and reliability of the analysis results due to the inadequate sample size; thus, we anticipate conducting further validations in larger patient cohorts. Secondly, the exploration is based on data mining analysis, although it has identified several potentially important findings, the lack of independent validation may weaken the results and their clinical application values; further research should incorporate wet lab experiments (such as using cell lines or clinical specimens) to ensure the accuracy and reproducibility of the results. Addressing these limitations will be crucial for advancing our understanding of cuproptosis-related TME in ccRCC and developing more effective treatments for this devastating disease.

## Conclusion

Collectively, we constructed a comprehensive scRNA-seq landscape of ccRCC, discovered the reduced cuproptosis activity along with the progress while recovered after therapy. Our results firstly revealed *HILPDA*+ ccRCC1 subtype based on scRNA-seq and ST-seq datasets, which exhibits heightened cuproptosis-susceptible characteristics with better prognosis, might interact with immunosuppressive cells to shape the cuproptosis-related immunosuppressive TME. The identification of the *HILPDA*+ ccRCC1 subtype provides novel insights into the role of cuproptosis in the immunosuppressive TME of ccRCC, and suggest potential cuproptosis-targeted therapy strategies for future clinical applications.

### Supplementary Information

Supplementary Material 1.

Supplementary Material 2.

Supplementary Material 3.

Supplementary Material 4.

Supplementary Material 5.

Supplementary Material 6.

## Data Availability

The scRNA-seq and ST-seq datasets used in this study were obtained from GEO database (GSE210038, GSE207493) and Mendeley Data platform (https://data.mendeley.com/datasets/g67bkbnhhg/1).
