# Supplementary material for: Characterization of cuproptosis signature in clear cell renal cell carcinoma by single cell and spatial transcriptome analysis
Source: Discov Oncol. 2024 Jul 24;15:300. doi: 10.1007/s12672-024-01162-2 (PMC11266328; doi:10.1007/s12672-024-01162-2)
Supplement: Supplementary file 1 — Supplementary Material 1. [file 12672_2024_1162_MOESM1_ESM.docx]

**Characterization of cuproptosis signature in clear cell renal cell carcinoma by single cell and spatial transcriptome analysis**

Xiaohong Zou^#^, Xiaoqing Liu^#^, Huiting Wang, Zhenhua Li, Chen Zhou

Department of Laboratory Medicine，The Eighth Affiliated Hospital，Sun Yat-Sen University，Shenzhen 518033， China

^#^Xiaohong Zou and Xiaoqing Liu contributed equally to this work.

Corresponding author:

Chen Zhou ([jacksmith1992@163.com](mailto:jacksmith1992@163.com))

**Figure S1 Quality control of integrated scRNA-seq dataset.**

**Figure S2 UMAP showing 7 main cell types in each sample.**

**Figure S3 Dot plot showing the enriched biological processes of stage-specific DEGs of each cell type.**

**Figure S4 Dot plot showing the relative expression levels of the top 5 cluster-specific genes of each cell type.**

**Figure S5 Bar plot showing the enriched biological processes of each Epithelial subtype.**

**Figure S6 UMAP plot of cell clustering across different stages.**
